# Pathway with single‐dose long‐acting intravenous antibiotic reduces emergency department hospitalizations of patients with skin infections

**DOI:** 10.1111/acem.14258

**Published:** 2021-05-05

**Authors:** David A. Talan, William R. Mower, Frank A. Lovecchio, Richard E. Rothman, Mark T. Steele, Katelyn Keyloun, Patrick Gillard, Ronald Copp, Gregory J. Moran

**Affiliations:** ^1^ Ronald Reagan UCLA Medical Center David Geffen School of Medicine at the University of California at Los Angeles Los Angeles CA USA; ^2^ Valleywise Health ASU University of Arizona and Creighton College of Medicine Phoenix Arizona USA; ^3^ Johns Hopkins Medical Center Johns Hopkins School of Medicine Baltimore Maryland USA; ^4^ Truman Medical Center University of Missouri–Kansas City School of Medicine Kansas City Missouri USA; ^5^ AbbVie Inc Irvine California USA; ^6^ ICON plc Dublin Ireland; ^7^ Olive View UCLA Medical Center David Geffen School of Medicine at the University of California at Los Angeles Los Angeles California USA

**Keywords:** abscess, antibacterial agents, cellulitis, critical pathways, dalbavancin, emergency department, hospital, health resources, hospitalization, skin diseases, infectious, wound infection

## Abstract

**Objectives:**

Emergency department (ED) patients with serious skin and soft tissue infections (SSTIs) are often hospitalized to receive intravenous (IV) antibiotics. Appropriate patients may avoid admission following a single‐dose, long‐acting IV antibiotic.

**Methods:**

We conducted a preintervention versus postintervention design trial at 11 U.S. EDs comparing hospitalization rates under usual care to those using a clinical pathway that included a single IV dalbavancin dose. We enrolled adults with cellulitis, abscess, or wound infection with an infected area of ≥75 cm^2^ without other indications for hospitalization. Clinical pathway participants discharged from the ED received a 24‐hour follow‐up telephone call and had a 48‐ to 72‐hour in‐person visit. We hypothesized that, compared to usual care, the clinical pathway would result in a significant reduction in the initial hospitalization rate.

**Results:**

Of 156 and 153 participants in usual care and clinical pathway periods, median infection areas were 255.0 (interquartile range [IQR] = 150.0 to 500.0) cm^2^ and 289.0 (IQR = 161.3 to 555.0) cm^2^, respectively. During their initial care, 60 (38.5%) usual care participants were hospitalized and 27 (17.6%) pathway participants were hospitalized (difference = 20.8 percentage points [PP], 95% confidence interval [CI] = 10.4 to 31.2 PP). Over 44 days, 70 (44.9%) usual care and 44 (28.8%) pathway participants were hospitalized (difference = 16.1 PP, 95% CI = 4.9 to 27.4 PP).

**Conclusions:**

Implementation of an ED SSTI clinical pathway for patient selection and follow‐up that included use of a single‐dose, long‐acting IV antibiotic was associated with a significant reduction in hospitalization rate for stable patients with moderately severe infections.

**Registration:** NCT02961764.

## INTRODUCTION

Emergency department (ED) visits for skin and soft tissue infections (SSTIs) increased almost threefold between 1993 and 2005.^1^ In 2009, approximately 870,000 patients were hospitalized for treatment of SSTIs in the United States,^2^ with an average length of stay (LOS) of 7.3 days and total cost of $4.84 billion.^3^ Several lines of evidence suggest that hospitalizations for SSTIs can be reduced.^4^, ^5^ In‐hospital mortality associated with SSTIs is less than 0.5%.^4^, ^6^ Mower at al.^7^ recently reported that among 2,923 patients seen in three EDs for SSTI, only 84 (2.9%) required intensive care unit (ICU) admission or operating room intervention or died. Talan et al.^8^ found that among patients with SSTIs presenting to a U.S. network of 11 U.S. EDs, administration of parenteral antibiotics was the only reason for hospital admission in approximately 40% of patients, suggesting that outpatient care would be feasible in many patients if parenteral antibiotic treatment could be provided. Recently, long‐acting parenteral antibiotics for treatment of SSTIs have been introduced that, as a single dose, can provide definitive treatment.^9^, ^10^, ^11^, ^12^, ^13^ An ED SSTI clinical pathway that could identify appropriate candidates for outpatient treatment following administration of a single‐dose, long‐acting intravenous (IV) antibiotic among patients who would otherwise be hospitalized could reduce hospital admissions and associated costs and improve patient satisfaction.^8^, ^14^, ^15^, ^16^, ^17^

The goal of this investigation was to determine if implementation of a clinical pathway that included use of a single‐dose, long‐acting IV antibiotic, dalbavancin, reduced the hospitalization rate for ED patients with more advanced SSTIs. These patients had more advanced infections consistent with the definition by the U.S. Food and Drug Administration (FDA) of an acute bacterial skin and soft tissue infection (ABSSSI; e.g., an area of infection of ≥75 cm^2^).^15^, ^18^ Therefore, we conducted a multicenter ED‐based preintervention versus postintervention pragmatic clinical trial that compared the hospitalization rate associated with usual care in the preintervention period to that following implementation of a clinical pathway in the postintervention period. We hypothesized that use of a clinical pathway that incorporated dalbavancin use would be associated with a significant reduction in the initial hospitalization rate.

## METHODS

### Design and setting

We conducted the ADVANCE (A Pragmatic TriAl Designed to eValuate a new Critical PAthway for Treatment of Patients with Acute Bacterial SkiN and Skin StruCture InfEctions, ADVANCE; ClinicalTrials.gov number, NCT02961764) trial at 11 U.S. academically affiliated EDs. This trial was sponsored by Allergan plc (Dublin Ireland; prior to its acquisition by AbbVie). The primary investigators (DAT, WRM, and GJM) designed the trial, had full access to the data, and vouch for the fidelity of the trial to the study protocol and statistical analysis plan and the accuracy of the results.

The institutional review board at each site approved the trial. Trial sites are described in the Data Supplement S1, Appendix 1 (available as supporting information in the online version of this paper, which is available at http://onlinelibrary.wiley.com/doi/10.1111/acem.14258/full). Attending emergency physicians supervised care at all sites. Prior to the study, sites did not have an existing SSTI clinical pathway or dalbavancin routinely available. Each site enrolled a group of patients in the usual care period and a separate group of patients in the clinical pathway period, which allowed each site to serve as its own control. During the usual care period, we only informed providers that patients with SSTI were being studied to determine practice patterns and associated outcomes; they were not informed of the planned clinical pathway intervention or the study objective to compare between‐period hospitalization rates.

### Selection of patients

Patients were assessed in the ED and enrolled based on the same inclusion and exclusion criteria in both the usual care (February 2017 to January 2018) and the clinical pathway periods (March 2018 to March 2019). Enrollment criteria were created based on investigator consensus to identify patients with a moderately severe SSTI for whom parenteral therapy could be indicated due to infection severity, yet who, based on the judgment of the treating providers, could otherwise receive treatment in an outpatient setting.^15^, ^16^ SSTIs were defined as skin lesions with erythema, swelling, tenderness, and/or drainage based on physical examination and categorized as follows: abscess was defined as a closed skin lesion found to have purulent exudate upon incision and drainage, cellulitis was defined as a closed skin lesion without evidence of a wound, and wound infection was defined as an open skin lesion. We estimated lesion area by measurement of the maximal length and perpendicular width of the infection, which was outlined by a tissue marker, using the formula for an ellipse (1/4 × π × length ×width).

We enrolled patients who were ≥18 years of age with abscess, cellulitis, or wound infection of known or suspected Gram‐positive etiology with an area of infection of ≥75 cm^2^. Exclusion criteria included patients with an unstable comorbidity (e.g., diabetic ketoacidosis, severe sepsis), immunosuppression, injection drug use and fever, pregnancy or breastfeeding, bilateral lower extremity involvement, severe neurological disorder, history of allergy to glycopeptide antibiotics, suspected Gram‐negative infection, infection likely to require broad‐spectrum antibiotics or more intensive care (e.g., infections associated with abdominal surgery, perirectal or perineal location, diabetic foot or decubitus ulcer, or animal or human bite or requiring drainage or debridement in the operating room or intensive care), known or suspected osteomyelitis, septic arthritis, or endocarditis (Appendix 1). These enrollment criteria are consistent with the U.S. FDA's definition of an ABSSSI,^18^ for which dalbavancin treatment has been FDA‐approved.^19^ We also required eligible patients to be willing to return for evaluation and provide informed consent. A complete description of the enrollment criteria can be found in Appendix 1.

### Baseline evaluation and interventions

During both the usual care and the clinical pathway periods, we collected baseline characteristics, including demographic and clinical findings, and previous health resource utilization data. We determined comorbidities to calculate the Charlson Comorbidity Index score.^20^

During the usual care period, participants were treated for SSTI based on usual care. During a 2‐ to 4‐week period between the end of the usual care and beginning of the clinical pathway period, each site's principal investigator and study coordinators trained physicians and other ED staff on the new clinical pathway. The clinical pathway included use of a single IV dose of dalbavancin over 30 minutes as initial empirical ED antibiotic treatment of each participant. We educated providers about dalbavancin use including drug characteristics, clinical trial results, dosing, associated adverse events (AEs), warnings, and contraindications based on the drug's package insert.^19^ We informed providers (and participants later) that dalbavancin would be provided free of charge. We dosed dalbavancin at 1500 mg for participants with creatinine clearance ≥ 30 mL/min and 1,125 mg for participants with creatinine clearance < 30 mL/min not receiving regularly scheduled dialysis.^19^ We also explained that participants discharged from the ED during the clinical pathway period would have a follow‐up by a telephone call at about 24 hours and a scheduled visit about 48 to 72 hours after ED discharge to evaluate their clinical response. Although dalbavancin was not in routine use at any site ED during the usual care period, its use was not prohibited. During the clinical pathway period, study coordinators reminded providers of the clinical pathway at the time of patient screening for enrollment.

During both periods, the decision to admit a participant for hospital care or discharge a participant from the ED for outpatient care was at the discretion of the treating provider; neither the study hypothesis nor encouragement to discharge patients was provided during the training or postintervention phase periods. During the usual care period, follow‐up care occurred but was at the discretion of the treating provider, whereas it was determined by protocol in the pathway period (see above). For participants unable to be seen back at the site, the study coordinator conducted a telephone interview. Study coordinators conducted subsequent evaluations in both time periods by telephone at 14 and 44 days after enrollment.

### Outcome measures

We assessed primary and secondary outcomes by participant interview and review of the site electronic medical record for subsequent hospitalization and other health resource use. The primary outcome was hospitalization rate at the time of initial care in the population that received at least one antibiotic dose (i.e., full analysis set [FAS] population). As a secondary outcome, we assessed hospitalizations through 44 days. We chose 44 days as the follow‐up period assuming a maximum treatment duration for the initial infection of 14 days and assessment of relevant outcomes over the subsequent 30 days. Furthermore, recurrent or new skin infections have been described to occur in about 10% to 20% of patients with these types of skin infections over this period of time.^21^, ^22^ For participants who did not have follow‐up contact through 44 days, we assumed no additional hospitalizations beyond the last follow‐up contact if review of their electronic medical records at the site hospital did not identify subsequent hospital admission.

Other secondary outcomes were evaluated in the FAS population, including health resource utilization (e.g., ED and inpatient LOS, level of care, major surgical interventions, ICU admissions, ED and other outpatient visits, hospitalizations), AEs, and patient‐related outcomes (e.g., a patient satisfaction survey, Work Productivity and Activity Impairment Questionnaire: Specific Health Problem V2.0 [WPAI:SHP],^23^ Health‐Related Quality of Life Medical Outcomes Study Short Form‐12 [SF‐12]).^24^ We administered study questionnaires including patient‐related outcome surveys 14 days after enrollment; the SF‐12 was also administered at baseline. Site investigators assigned hospital days and other health care events as infection‐related or not and AEs as antibiotic‐related or not.

### Data analysis

We designed our trial as a superiority trial. Our primary hypothesis was that, compared with usual care, implementation of a SSTI clinical pathway, which included use of a single‐dose of a long‐acting IV antibiotic, would be associated with significant reduction in the initial hospitalization rate. We assumed an effect size of a 15‐percentage‐points (PP) decrease from an initial hospitalization rate of 45%. This effect size was based on estimates of the rate of hospitalization for IV antibiotics only for patients with characteristics of this study population^8^ and the smallest clinically meaningful difference. With an 80% power and alpha of 0.05, we estimated the required sample size to be 322 participants (161 participants in each period), which was confirmed after a planned interim analysis once 75 participants were enrolled on usual care. The estimated final enrollment included adjustments for attrition that supported one‐sided hypothesis testing (two‐sample Pearson chi‐square test for proportion difference with equal group weights using a normal approximation); this hypothesis assumed that the hospital admission rates comparing the preintervention and postintervention periods were 38% and 23%, respectively. A one‐sided analysis was conducted based on the assumption that introduction of a single‐dose long‐acting parenteral antibiotic could only decrease the hospitalization rate among patients who previously had only been admitted to receive IV antibiotics. Based on results of a planned interim analysis that was designed to check the sample size assumptions after approximately 75 usual care patients were enrolled, an adjustment was made at the time of this interim analysis (and prior to completion of the usual care period) to revise the primary outcome from total admitted hospital days to hospital admission rate at the initial episode of care at the time of the index ED visit. This change was made because a primary outcome of total admitted days was projected to require a much larger sample size and study duration than would have been feasible (interim analysis estimated over 10 times the sample size initially planned).

To test the study hypothesis, we compared hospitalization rates in the two periods using the PP difference and 95% confidence intervals (CIs) of the difference. We defined superiority to exist if the lower limit of the CI for the difference in the admission rates between usual care and the clinical pathway exceeded zero. We presented secondary outcomes in the FAS population by descriptive analyses with PP differences and 95% CIs of the differences. We performed descriptive analyses on categorical and continuous variables. Results of these descriptive assessments are presented in terms of proportions and CIs (categorical data), and medians and interquartile ranges (IQRs; continuous data). To assess the potential for confounding factors, we conducted an adjusted multivariate logistic regression analysis predicting hospital admission using stepwise selection (threshold of p < 0.1) from a predetermined list of variables identified a priori due to clinical relevance. Our final variables for adjustment included age, race, insurance type, prior resource use (no/yes), and systemic inflammatory response syndrome (SIRS) score (<2 vs. ≥2). Finally, we conducted a time‐series analysis based on the weekly proportion of patients admitted to assess the potential effects that temporal factors, including trends (assessed using the runs test) and autocorrelation (one‐lag apart autocorrelation values and Durbin‐Watson statistic), might play in admission rates.

## RESULTS

### Characteristics of study participants

**FIGURE 1:**
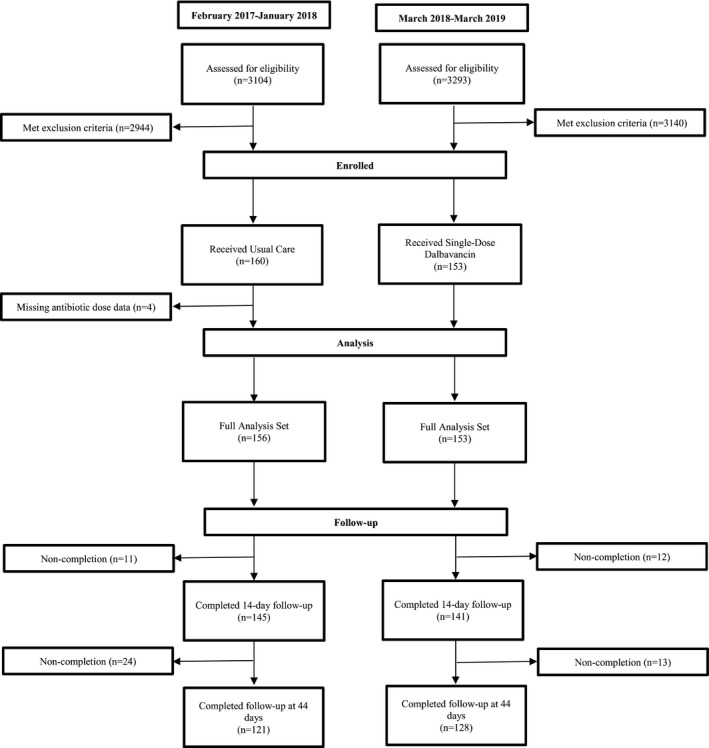
During the usual care and clinical pathway periods, patients were selected if they fulfilled eligibility requirements, i.e., adults with cellulitis, abscess, or wound infection with an infected area of ≥75 cm^2^ and a known or suspected Gram‐positive infection without other indications for hospitalization (e.g., unstable coorbidities, requiring the operating room or intensive care). In the usual care period, participants were treated for SSTI based on usual care. Once the usual care period was completed, over 2 to 4 weeks prior to the initiation of the clinical pathway period, each site's principal investigator and study coordinators trained physicians and other ED staff on the clinical pathway. During the clinical pathway period, all participants were administered a single IV dose of dalbavancin in the ED. For participants who did not have follow‐up contact through 44 days, it was assumed that there were no additional hospitalizations beyond the last follow‐up contact if review of their electronic medical records at the site hospital did not identify subsequent hospital admission. SSTI, skin and soft tissue infection

Patient screening, enrollment, and follow‐up are described in Figure 1. Of 3,104 patients presented to site EDs with SSTI screened during the usual care period and 3,293 patients screened during the clinical pathway period, 160 (5.2%) and 153 (4.7%) participants were enrolled, respectively. Four enrolled patients during the usual care period were missing information regarding antibiotic dose and were excluded. The study population consisted of 156 participants on usual care and 153 participants on the clinical pathway. Of these, 145 (92.9%) and 141 (92.2%) were followed through 14 days, and 121 (77.6%) and 128 (83.7%) were followed through 44 days in the usual care and clinical pathway time periods, respectively.

Demographic and baseline clinical characteristics among participants in the usual care and pathway periods were similar (Table 1 **)**. Among all 309 participants in the study population, 64% were male. Median age was 47.5 (IQR = 32.0 to 58.5) years for participants on usual care and 46.0 (IQR = 33.0 to 58.0) years on the clinical pathway. Median infection area was 255.0 (IQR = 150.0 to 500.0) cm^2^ for participants on usual care and 289.0 (IQR = 161.3 to 555.0) cm^2^ on the clinical pathway. Overall, 58 (18.8%) participants exhibited two or more SIRS criteria. Diabetes was the most common comorbidity and was present in 18.8% of participants in each group. The most common type of infection was cellulitis, diagnosed in 81.9% of participants.

**TABLE 1:** Characteristics of participants treated preimplementation and postimplementation of a SSTI clinical pathway

Characteristic	Usual care (*n* = 156)	Clinical pathway (*n* = 153)
Age (y)		
Median (Q1, Q3)	47.5 (32.0, 58.5)	46.0 (33.0, 58.0)
Range	19.0, 94.0	18.0, 97.0
Male, n (%)	100 (64)	99 (65)
Ethnicity, n (%)		
Not Hispanic/Latino	116 (74.4)	107(69.9)
Hispanic/Latino	39 (25.0)	43 (28.1)
Unknown	1 (0.6)	3 (2.0)
Race, n (%)		
White	90 (57.7)	91 (59.5)
Black	30 (19.2)	39 (25.5)
Asian	1 (0.6)	2 (1.3)
Other	35 (22.4)	21 (13.7)
Admitted to ED from location, n (%)		
Private home/independent senior living facility	144 (92.3)	132 (86.3)
Long‐term care or skilled nursing facility, nursing home, or rehab facility	5 (3.2)	5 (3.3)
Hospital	0	5 (3.3)
Other/unknown	7 (4.5)	11 (7.2)
Primary insurance, n (%)		
Private commercial plan	42 (26.9)	33 (21.6)
Government funded	84 (53.8)	89 (58.2)
Uninsured	25 (16.0)	30 (19.6)
Other/unknown	5 (3.2)	1 (0.7)
Work status, n (%)		
Employed	81 (51.9)	73 (47.7)
Unemployed	65 (41.7)	80 (52.3)
Unknown	10 (6.4)	0
Infection type, n (%)		
Cellulitis/erysipelas	127 (81.4)	126 (82.4)
Abscess	51 (32.7)	55 (35.9)
Wound infection	15 (9.6)	9 (5.6)
No purulent drainage, n (%)	118 (75.6)	119 (77.8)
Primary lesion size (cm^2^)		
Median (Q1, Q3)	255.0 (150.0, 500.0)	289.0 (161.3, 555.0)
Range	75.0, 196.0	77.0, 3905.5
Primary lesion location, n (%)		
Upper/lower leg	88 (56.4)	82 (53.6)
Upper/lower arm	27 (17.3)	28 (18.3)
Torso	21 (13.5)	22 (14.4)
Foot	12 (7.7)	16 (10.5)
Hand	5 (3.2)	3 (2.0)
Head/neck	3 (1.9)	2 (1.3)
SIRS criteria (≥2), n (%)^a^	31 (19.9)	27 (17.6)
Fever	11 (7.1)	5 (3.3)
Heart rate	71 (45.5)	63 (41.2)
Respiration	5 (3.2)	1 (0.7)
White blood cell count	46 (29.5)	35 (22.9)
BMI (kg/m^2^)		
Median (Q1, Q3)	31.9 (25.0, 40.5)	29.4 (24.8, 39.2)
Range	15.1, 60.6	18.0, 77.9
Comorbid conditions, n (%)		
Diabetes mellitus	27 (17.3)	31 (20.3)
Injection drug use	13 (8.3)	19 (8.5)
Liver disease	9 (5.8)	9 (5.9)
Chronic obstructive pulmonary disease	7 (4.5)	7 (4.6)
Congestive heart failure	6 (3.8)	5 (3.3)
Lymphedema/chronic venous stasis	6 (3.8)	2 (1.3)
Myocardial infarction	6 (3.8)	5 (3.3)
Solid tumor	6 (3.8)	3 (2.0)
Cerebrovascular disease	5 (3.2)	5 (3.3)
Peripheral vascular disease	5 (3.2)	4 (2.6)
Moderate to severe chronic kidney disease	4 (2.6)	5 (3.3)
Malignant lymphoma	3 (1.9)	1 (0.7)
Peptic ulcer disease	3 (1.9)	1 (0.7)
Connective tissue disease	1 (0.6)	2 (1.3)
Dementia	1 (0.6)	1 (0.7)
Leukemia	1 (0.6)	0
AIDS	0	1 (0.7)
Charlson Comorbidity Index		
Median (Q1, Q3)	0.96 (0.90, 0.98)	0.96 (0.90, 0.98)
Range	0.0, 0.98	0.0, 0.98
Immunocompromising conditions, n (%)		
HIV	3 (1.9)	3 (2.0)
Organ transplant recipient	1 (0.6)	1 (0.7)
Received TNF inhibitors	0	0
Received any other immune modulating medication, including biologics	1 (0.6)	0
Received chemotherapy	1 (0.6)	1 (0.7)

Abbreviations: BMI, body mass index; HIV, human immunodeficiency virus; Q1, 25th percentile; Q3, 75th percentile; SIRS, systemic inflammatory response syndrome; SSTI, skin and soft tissue infection; TNF, tumor necrosis factor.

^a^ Patients met SIRS criteria if they had ≥2 of the following criteria: temperature > 38.0°C or < 36.0°C, heart rate > 90 beats/min, respiratory rate > 20 breaths/min, or white blood cell count >12,000/µL or <4,000/µL or >10% band neutrophils.

Of 149 participants receiving usual care and having antibiotic data available, 41 (27.5%) were initially treated with an IV antibiotic in the ED prior to discharge, with 39 of these 41 participants discharged on oral antibiotics. Of 89 participants discharged from the ED, 47 (52.8%) received only oral antibiotics. One participant was treated with antibiotic ointment and received neither IV nor oral antibiotics. The most common IV antibiotics given in the ED were vancomycin (42.3%), cefazolin (21.2%), and ceftriaxone (10.3%), and the most common oral antibiotics prescribed were cephalexin (38.5%), clindamycin (35.3%), trimethoprim‐sulfamethoxazole (30.1%), and doxycycline (9.6%). Of 153 participants on the clinical pathway, 152 (99.3%) were initially treated with IV dalbavancin administered in the ED (one participant received dalbavancin after ED discharge). Of these participants, 46 (30.0%) were also given oral antibiotics at the time of ED discharge. The most common oral antibiotics prescribed were cephalexin (13.1%), trimethoprim‐sulfamethoxazole (13.1%), and clindamycin (5.9%).

### Main results

Over 14 days, or during their initial care, 60 (38.5%) usual care participants were hospitalized and 27 (17.6%) pathway participants were hospitalized (difference = 20.8 PP, 95% CI = 10.4 to 31.2 PP). Median all‐cause hospital LOS during the initial visit was 3.0 (IQR = 2.0 to 5.0, range = 0 to 16) days among admitted usual care participants and 2.0 (IQR = 1.0 to 4.0, range = 0 to 14) days among admitted postintervention period participants. Over 14 days, or during their initial care, usual care participants received a cumulative total of 207 days of hospitalization or 1.3 days for each enrolled participant (3.5 days per admitted participant), while clinical pathway participants received a cumulative total of 100 days of hospitalization or 0.7 days for each enrolled participant (3.7 days per admitted participant). Sensitivity analyses, assuming that all participants lost to follow‐up were either hospitalized or not admitted, are provided in Appendix 2.

Over 44 days, the hospitalization rates in the usual care and pathway groups were 44.9% and 28.8%, respectively (difference = 16.1 PP, 95% CI = 4.9 to 27.4 PP). Median LOS for subsequent admissions was 3.0 (IQR = 1.0 to 4.5, range = 0 to 31) days among usual care participants and 3.0 (IQR = 1.0 to 4.0, range = 1 to 7.0) days among clinical pathway participants. Following initial care and ED or hospital discharge, 18 (11.5%) usual care participants were hospitalized, including readmission of four participants who were admitted upon their initial presentation. During this period, 20 (13.1%) clinical pathway participants were admitted, including four readmissions for three participants who were admitted upon their initial presentation. For subsequent hospitalizations, usual care participants received a cumulative 99 hospital days, while clinical pathway participants received a cumulative total of 59 days. Over 44 days, infection‐related ED visits occurred in 22 (14.1%) usual care cases and 19 (12.4%) pathway participants. Infection‐related outpatient visits occurred in 57 (36.5%) of usual care participants and 39 (25.5%) of pathway participants. Over 44 days, infection‐related surgery requiring the operating room occurred in one (0.6%) of usual care and five (3.3%) of pathway participants, and ICU admission occurred in three (1.9%) of usual care and one (0.7%) of pathway participants. No participant died from an infection‐related or antibiotic‐related cause.

Results from the multivariate logistic regression produced an admission odds ratio of 0.289 (95% CI = 0.156 to 0.532, p < 0.001) similar to results of our unadjusted admission rate comparisons, in which patients in the postintervention period were significantly less likely to be admitted when compared with patients enrolled in the preintervention period.

Our time‐series analysis found no evidence of trend in the usual care (runs test = 25, p = 0.44) or pathway care (runs test = 21, p = 0.24), but did reveal suggestive evidence of trend through the combined periods (runs test = 34, p = 0.07). Our assessments also revealed no evidence of autocorrelation during usual care (one‐lag‐apart autocorrelation = –0.14, Durbin‐Watson statistic = 1.08) or pathway care (one‐lag‐apart autocorrelation = –0.07, Durbin‐Watson statistic = 1.53) or across the combined periods (one‐lag‐apart autocorrelation = 0.03, Durbin‐Watson statistic = 1.16).

Most AEs were mild or moderate in severity. Among all AEs, those characterized as mild occurred in eight (5.2%) usual care and 48 (31.4%) clinical pathway participants. None of the AEs led to study discontinuation and all patients recovered. AEs categorized as possibly antibiotic‐related occurred in six (3.8%) usual care and 25 (16.3%) clinical pathway participants. Moderate and severe AEs occurred in eight (5.1%) and six (3.8%) usual care participants and 12 (7.8%) and eight (5.2%) clinical pathway participants, respectively. The most common AEs categorized as possibly antibiotic‐related were diarrhea (1.3%) and nausea (1.3%) on usual care and diarrhea (3.9%), nausea (2.0%), and skin and subcutaneous tissue disorders (4.6%) on the clinical pathway. The majority of severe AEs were related to SSTI progression (usual care, 3.2%; clinical pathway, 9.2%; serious AE results are presented in the Appendix 2). One clinical pathway participant developed *Clostridiodes difficile* colitis.

Patient‐related outcomes are reported in the Appendix 2. When surveyed at 14 days, most participants in each period indicated they were satisfied with their ED wait time, hospital stay (when applicable), and IV antibiotic therapy. Most participants in each period indicated they would prefer outpatient care if treated again. The WPAI:SHP questionnaire supported a similar impact of skin infection and its treatment on participant work impairment between trial periods. Among 58 usual care respondents, 19 (32.8%) reported missed work days related to their illness compared to 18 of 54 (33.3%) clinical pathway respondents (difference = 0.6%, 95% CI = –18.6 to 19.8) and 19 of 53 (35.8%) usual care respondents reported impairment with work‐related activities as compared with 13 of 48 (27.1%) clinical pathway respondents (difference = –8.8%, 95% CI = –28.8 to 11.2). In addition, 55 of 121 (45.5%) usual care respondents indicated impairment in non–work‐related activities compared with 34 of 123 (27.6%) clinical pathway respondents (difference = –17.8%, 95% CI = –30.5 to –5.1**)**. Both the mental and the physical component summary scores increased between baseline and Day 10 to 14 visit in usual care and clinical pathway participants, supporting an improvement in quality of life.

## DISCUSSION

In this preintervention versus postintervention period design trial among adults presenting to EDs with more advanced SSTI (median area = approximately 250–300 cm^2^) who were otherwise candidates for outpatient care, implementation of a clinical pathway that included use of a single‐dose long‐acting IV antibiotic, dalbavancin, and a follow‐up telephone call at 24 hours and visit at 48 to 72 hours, was associated with a significant reduction in all‐cause initial hospitalization rate compared to usual care of over 50%, from 38.5% to 17.6%, without increased subsequent ED and office visits. To the best of our knowledge, this is the first study to support the potential for single‐dose parenteral therapy to substantially reduce the hospitalization rate for patients with serious infections. The reduction of hospitalization rate once parenteral and adherent antibiotic treatment could be insured is consistent with the past observation of providers indicating that administration of IV antibiotics is the sole reason for hospitalization in approximately 40% of ED patients admitted for SSTI treatment.^8^ Since hospital stay is the major driver of total health care expenditures, strategies promoting outpatient management are potentially cost saving. When surveyed, our participants preferred avoiding hospitalization if possible.

In addition to reduction of total hospital admissions (i.e., initial hospitalization and readmission over 44 days), the safety of this clinical pathway was further supported by similar rates of moderate, severe, and serious AEs. Dalbavancin use was associated with increased incidence of mild AEs, which may have been related to knowledge of dalbavancin's prolonged duration compared with other antibiotics used during usual care. When dalbavancin was compared to IV and oral linezolid in a randomized, double‐blind trial, AE rates, including for mild AEs, were similar.^10^

Clinical pathways have been successfully employed to safely reduce ED hospitalization for other infections. In a cluster‐randomized trial of adults presenting to EDs with community‐acquired pneumonia, Marrie et al.^25^ demonstrated that risk stratification using the Pneumonia Severity Index (PSI) and levofloxacin treatment reduced the initial hospitalization rate of low‐risk participants from 49% to 31% with no difference in quality of life scores compared to those managed by usual care. Although a prospectively validated risk stratification model like the PSI does not yet exist for patients with SSTI, the relative risk of serious complications and death compared to patients with community‐acquired pneumonia suggests that an opportunity exists to substantially reduce these hospitalizations. In a retrospective analysis of 2,923 patients seen in three EDs for SSTI, Mower et al.^7^ recently found that one or more of six high‐risk variables (i.e., abnormal imaging [e.g., gas, abscess, osteomyelitis], systemic inflammatory response syndrome, diabetes, prior SSTI at the same location, age > 65 years, and hand location) was present among all of only 84 (2.9%) patients who required ICU admission or operating room intervention or died. In the current trial, we did not impose a maximum size of the infected area but generally excluded patients with unstable comorbidities or who were anticipated to require ICU care (e.g., severe sepsis) or surgery in the operating room (e.g., necrotizing fasciitis). Participants had very low rates of operating room surgery and ICU care, and there were no infection‐related deaths. Patient selection was a key component of the clinical pathway; however, it is important to note that many ED patients hospitalized for SSTIs lacked these types of exclusion criteria.^8^

Another approach to reducing avoidable hospitalization for infections are strategies to delivery IV treatments in the outpatient setting. Outpatient parenteral antibiotic treatment has been accomplished both through use of peripherally inserted central catheters and through retention of ED‐placed peripheral IV catheters with follow‐up by a visiting nurse or in a clinic for daily antibiotic administration.^16^, ^26^ The relatively recent introduction of single‐dose IV antibiotic treatments for patients with a SSTI‐infected area of >75 cm^2^, referred to as ABSSSI, ensures that the patient receives not only parenteral treatment but also a full course of treatment. ED patients often are challenged to have good adherence to an oral antibiotic regimen or lack resources that promote a care strategy that requires frequent follow‐up visits. Further evaluation SSTI risk stratification schemes in conjunction with clinical pathways, such as the one evaluated in this trial, in other populations and settings will help optimize care strategies for patients with SSTI.

## LIMITATIONS

This trial has limitations. Participants were not randomized to usual treatment or care guided by the clinical pathway with dalbavancin use. While randomization would have addressed some potential confounders, such as those related to differences in providers, participants, and practices, as well as other secular trends between the usual care and the clinical pathway periods, it could have also resulted in provider awareness of being studied and learning from the experimental approach affecting usual care. Cluster randomization, which might have avoided cross‐contamination of approaches, would also have been limited by potential imbalances between groups of sites.

We chose a preintervention versus postintervention period design, which allowed each site to serve as its own control. Participant demographics and baseline clinical characteristics were similar in the usual care and clinical pathway periods. This was a pragmatic study, so treating providers and site investigators were not blinded to the patient's care. While the assurance of parenteral antibiotic coverage with dalbavancin likely resulted in a low hospitalization rate, the clinical pathway also differed from usual care in that a 24‐hour telephone call and 48‐ to 72‐hour follow‐up visit were prescribed as opposed to visits left to clinician discretion, which were not restricted. While we did not record the recommended follow‐up in the usual care period, participants had more infection‐related ED and other outpatient visits than the pathway period participants. This study did not assess whether participants derived some outcome benefit from IV antibiotics compared to oral antibiotic treatment^27^ or hospital compared to outpatient care. Approximately 8% of participants were lost to follow‐up at 14 days. The sensitivity analysis supported pathway reduction of hospitalization and assumed that all missed cases were admitted, but did not support the assumption that all usual care participants were not admitted and all pathway participants were admitted. The assumption of the latter condition is much less likely. There may also be unaccounted temporal factors that could have affected the differences in admission rates we observed between the period of usual care and pathway care and that were not accounted for or evident in our time series analysis.

Since dalbavancin use was not standard care at sites, we felt obligated to provide it free of charge to participants. While this allowed us to better isolate the extent to which a provider's decision to hospitalize a patient with a more advanced SSTI was related to the perceived need for IV treatment or to guarantee antibiotic adherence, it also eliminated this drug's cost as part of decision making. The 2019 wholesale acquisition cost for 1,500 mg of dalbavancin was $4,604,^28^ whereas the estimated cost of hospital stay in 2017 was $2,424/day; the median LOS during the usual care period was 3.0 days.^29^ Although dalbavancin was not routinely available to sites during the usual care period, it could be used and was provided to two participants in this period. Thirty percent of participants receiving dalbavancin also had an oral antibiotic prescribed, which likely reflected some providers’ misunderstanding about the drug's activity or duration of action, but this should not have affected outcomes for presumed Gram‐positive SSTI.

In retrospect, we wish we had better tracked the specific reasons for patient exclusion. Our group previously found that about 15% of ED patients presenting with SSTI were hospitalized and by far the most common reason for admission was IV antibiotics (85%), which was the sole reason in about 40%.^8^ Therefore, it is conceivable that about one‐third of those intended to be hospitalized, or about 5% of SSTI patients overall, the proportion of SSTI patients we enrolled, might qualify for outpatient care following single‐dose parenteral antibiotic treatment. Thus, we believe that our findings likely apply to most adults with SSTI with an area of infection of ≥75 cm^2^, but these results should not be applied to other patients who have any of the conditions described in the study's exclusion criteria.

## CONCLUSIONS

Implementation of an ED skin and soft tissue infection clinical pathway for patient selection and follow‐up that included use of a single‐dose, long‐acting IV antibiotic was associated with a significant reduction in hospitalization rate for stable patients with moderately severe infections.

## CONFLICT OF INTEREST

DAT has received consulting fees from AbbVie Inc., GSK, and Spero. GJM has received consulting fees from AbbVie Inc. and funding for clinical research from Cempra, Contrafect, and Nabriva. WRM, FAL, RER, and MTS received consulting fees from AbbVie Inc. KK and PG are employees of AbbVie Inc. and may have AbbVie stock. RC was an employee of ICON plc, the CRO supporting study conduct.

## AUTHOR CONTRIBUTIONS

All authors met the ICMJE authorship criteria. All authors conceived and designed the protocol and developed the methodology. All authors played a role in the trial design; collection, management, analysis, and interpretation of data; writing of the report; and the decision to submit the report for publication. All authors directed development of the manuscript and reviewed and commented on all manuscript drafts. All authors read and approved the final manuscript. Specific author roles in the study are described in Appendix 1.

## Supporting information

Data Supplement S1. Supplemental material.
