# Supplementary material for: Pathway with single‐dose long‐acting intravenous antibiotic reduces emergency department hospitalizations of patients with skin infections
Source: Acad Emerg Med. 2021 May 5;28(10):1108–17. doi: 10.1111/acem.14258 (PMC8597095; doi:10.1111/acem.14258)
Supplement: Supplementary file 1 — Data Supplement S1. Supplemental material. [file ACEM-28-1108-s001.pdf]

Clinical Pathway With Single-Dose Long-Acting Intravenous Antibiotic Reduces Emergency Department Hospitalizations of Patients With Skin Infections

David A. Talan, MD, William R. Mower, MD, PhD, Frank A. Lovecchio, DO,
Richard E. Rothman, MD, PhD, Mark T. Steele, MD, Katelyn Keyloun, PharmD, MS,
Patrick Gillard, PharmD, MS, Ronald Copp, MPH, Gregory J. Moran, MD

Supplemental Appendix

Contents

APPENDIX 1. INCLUSION/EXCLUSION CRITERIA 2

APPENDIX 2. SENSITIVITY ANALYSIS 6

TABLE S1. INVESTIGATORS AND SITES 8

TABLE S2. SERIOUS ADVERSE EVENTS AMONG PARTICIPANTS TREATED PRE- AND POST- IMPLEMENTATION OF A SKIN AND SOFT TISSUE INFECTION CLINICAL PATHWAY 11

TABLE S3. PARTICIPANT SATISFACTION SURVEY RESULTS 13

TABLE S4. PARTICIPANT-REPORTED WORK PRODUCTIVITY AND ACTIVITY IMPAIRMENT ON USUAL CARE AND AFTER IMPLEMENTATION OF A SKIN AND SOFT TISSUE INFECTION CLINICAL PATHWAY 16

TABLE S5. PATIENT-REPORTED HEALTH-RELATED QUALITY OF LIFE OUTCOMES ON USUAL CARE AND AFTER IMPLEMENTATION OF A SKIN AND SOFT TISSUE INFECTION CLINICAL PATHWAY 17

Appendix 1. Inclusion/Exclusion criteria

Inclusion criteria

Eligible study subjects satisfied all of the following inclusion criteria:

- Age ≥18 years;
- Presented to the ED and met FDA clinical definition for acute bacterial skin and skin structure infection (ABSSSI);
- Presented with the following infection types: cellulitis/erysipelas, wound infection, or major cutaneous abscess;
- Lesion size area requirements, as measured manually by the length multiplied by perpendicular width of wound;
    - Lesion size area ≥75 cm^2;
- Known or suspected infection caused by susceptible isolates of the following Gram-positive microorganisms: *Staphylococcus aureus* (including methicillin-susceptible and methicillin-resistant strains), *Streptococcus pyogenes*, *Streptococcus agalactiae*, *Streptococcus dysgalactiae*, *Streptococcus anginosus* group (including *S. anginosus, S. intermedius, S. constellatus*) and *Enterococcus faecalis* (vancomycin susceptible strains); and
- Willing and able to return to the hospital or a designated clinic for scheduled visits, or be in contact with the study coordinator through telephone communication, as required by the protocol and the antibiotic treatment administered.

Exclusion criteria

Study subjects with any of the following criteria were excluded:

- History of allergy to glycopeptide antibiotics (eg, vancomycin, dalbavancin, oritavancin, telavancin, teicoplanin), excluding infusion-related reactions that were resolved through increasing the duration of the IV antibiotic infusion (eg, Red man syndrome);
- Known or suspected Gram-negative infections, including bacteremia, anaerobic infections, or fungemia, even in the presence of Gram-positive infection;
- Known or suspected infections that were severe, life-threatening, or not included in the ABSSSI FDA guidance, including the following examples:
    - Gangrene;
    - Known or suspected necrotizing fasciitis;
    - Known or suspected osteomyelitis, septic arthritis, or endocarditis;
    - Diabetic foot infection in presence of an ulcer (eg, cellulitis without ulcer on the foot of a diabetic patient is not means for exclusion); and
    - Decubitus or ischemic ulcer;
- Infections that likely required therapy with multiple antibiotics or more intensive care/observation, including the following examples:
    - Infection due to abdominal surgery;
    - Post-operative wound infection, with the exception of skin and soft tissue laceration repair;
    - Perirectal or perineal infections;
    - Infections that required drainage or debridement in the operating room;
    - Facial infections including dental, orbital, or sinus infections; and
    - Animal or human bites;

- Unstable comorbidity at ED presentation, including the following examples:
  - Severe sepsis (defined as sepsis-induced tissue hypoperfusion or organ dysfunction thought to be due to the infection, including: sepsis-induced hypotension; lactate above upper limits laboratory normal; urine output <0.5 mL/kg/hr for longer than 2 hours despite adequate fluid resuscitation; acute lung injury with PaO_2/FIO_2 <250 in the absence of pneumonia as infection source; acute lung injury with PaO_2/FIO_2 <200 in the presence of pneumonia as infection source; creatinine >2.0 mg/deciliter [dL] (176.8 micromoles [μmol]/liter [L]); bilirubin >2 mg/dL (34.2 μmol/L); platelet count <100,000 microliters (μL); coagulopathy (international normalized ratio >1.5) or septic shock (defined as sepsis-induced hypotension persisting despite adequate fluid resuscitation);
  - Hemodynamic instability requiring pharmacologic or mechanical support to maintain a normal blood pressure or adequate cardiac output; and
  - Active immunocompromised/profound immunosuppression including participants with underlying conditions such as protein-calorie malnutrition and uncontrolled diabetes (diabetic ketoacidosis or abnormalities in blood osmolarity), organ transplant recipients, participants with hematological malignancies, participants receiving therapeutic immunosuppression, and participants with AIDS (CD4 cell count <200 cells/mm^3), severe neutropenia (defined as an absolute neutrophil count [absolute neutrophil count <500/mm^3]);
- Injection drug users presenting with a fever;

- Positive urine (or serum) pregnancy test at screening, currently breastfeeding or females of childbearing potential who are unwilling or unable to use adequate contraceptive precautions (including abstinence) until the final study visit;
- Severe neurological disorder leading to severe immobility or confined to wheelchair or bed (eg, paraplegia, hemiplegia);
- Bilateral lower extremity involvement of the suspected infection (to exclude patients with chronic venous stasis); and
- Unwilling or unable to follow study procedures

Appendix 2. Sensitivity analysis

Sensitivity Analysis 1: Missing cases at 14 days considered hospitalized.

In the original analysis, data were available for 145 usual care participants (11 missing cases), of whom 69 were admitted in the first 14 days. There were 141 pathway participants (12 missing cases) with 14-day follow-up data, of whom 39 were admitted in the first 14 days. Assigning missing cases as hospitalized would result in admission for 80 participants in usual care (80 = 69 + 11), or 51.3% of usual care cases, as compared to admission for 51 pathway participants (51 = 39 + 12), or 33.3% of pathway participants. The difference in admission rates was 51.3% − 33.3% = 17.9% (95% CI: 6.5% to 29.4%). The difference significantly favors pathway care and the initial findings are robust under this assumption.

Sensitivity Analysis 2: Missing cases at 44 days are assigned not hospitalized in usual care participants and hospitalized in pathway participants.

In the original analysis, data were available for 121 usual care participants (35 missing cases), of whom 70 were admitted in first 44 days. There were 128 pathway participants (25 missing cases) with 44-day follow-up, of whom 44 were admitted in the first 44 days. Assigning missing cases in the usual care groups as not requiring hospitalization would result in 74 admissions among usual care participants (70 = 70 + 0), or 44.9% of usual care cases. Assigning missing cases as hospitalized in the pathway participants would

result in 69 admissions among pathway participants (69 = 44 + 25), or 45.1% of pathway participants. The difference in admission rates was 44.9% − 45.1% = −0.2% (95% CI: −12.0% to 11.5%). This difference was not significant, and the initial findings were not robust to this type of sensitivity analysis.

Table S1. Investigators and Sites

Investigator, Role, and Department
David Talan, MD<br>Principal Investigator<br>Emergency Medicine/Infectious Diseases Specialist<br>Department of Emergency Medicine and Medicine,<br>Division of Infectious Diseases<br>Ronald Reagan UCLA Emergency Medicine Center<br>Los Angeles, CA
Gregory J Moran, MD<br>Site Investigator<br>Emergency Medicine/Infectious Diseases Specialist<br>Department of Emergency Medicine and Medicine,<br>Division of Infectious Diseases<br>Olive View-UCLA Medical Center<br>Sylmar, CA
William R. Mower, MD, PhD<br>Site Investigator<br>Emergency Medicine Specialist and Biostatistician<br>Department of Emergency Medicine<br>Ronald Reagan UCLA Emergency Medicine Center<br>Los Angeles, CA
Mark Steele, MD<br>Site Investigator<br>Emergency Medicine Specialist<br>Department of Emergency Medicine<br>Truman Medical Center<br>Kansas City, MO
Richard Rothman, MD, PhD<br>Site Investigator<br>Emergency Medicine Specialist,<br>Department of Emergency Medicine<br>Johns Hopkins Hospital<br>Baltimore, MD
Frank Lovecchio, DO<br>Site Investigator<br>Emergency Medicine Specialist

Department of Emergency Medicine and Medical
Toxicology
Maricopa Medical Center
Phoenix, AZ

Joseph Kuti, PharmD
Site Investigator
Pharmacist
Hartford Hospital
Hartford, CT

John Haran, MD
Site Investigator
Emergency Medicine Specialist
Department of Emergency Medicine
University of Massachusetts Medical Center
Worcester, MA

Gary Peksa, PharmD, BCPS
Site Investigator
Pharmacist
Department of Pharmacy
Rush University Medical Center
Chicago, IL

Robert Sherwin, MD
Site Investigator
Emergency Medicine Specialist
Department of Emergency Medicine
Wayne State University
Detroit, MI

Howard Klausner, MD
Site Investigator
Emergency Medicine Specialist
Department of Emergency Medicine
Henry Ford Hospital
Detroit, MI

Vincent Rimanelli, DO
Site Investigator
Internal Medicine Specialist
Department of Internal Medicine/Emergency
Medicine
Promedica Monroe Regional Hospital
Monroe, MI

David Wein, MD
Site Investigator
Emergency Medicine Specialist
Department of Emergency Medicine
Tampa General Hospital
Tampa, FL

Table S2. Serious AEs Among Participants Treated Pre- and Post-Implementation of a Skin and Soft Tissue Infection Clinical Pathway

Category, n (%)	Usual Care* (n=156)	Clinical Pathway* (n=153)
Serious AEs	11 (7.1)	16 (10.0)
Infections and infestations	5 (3.2)	14 (9.2)
Cellulitis	2 (1.3)	8 (5.2)
Skin infection	2 (1.3)	0
Bronchitis	1 (0.6)	0
Abscess	0	1 (0.7)
Bacteremia	0	1 (0.7)
Clostridiodes difficile colitis	0	1 (0.7)
Lymphangitis	0	1 (0.7)
Skin bacterial infection	0	1 (0.7)
Subcutaneous abscess	0	1 (0.7)
Respiratory, thoracic and mediastinal disorder	2 (1.3)	1 (0.7)
Acute respiratory failure	1 (0.6)	0
Chronic obstructive pulmonary disease	1 (0.6)	0
Pulmonary embolism	0	1 (0.7)
Treatment failure	2 (1.3)	0
Anaphylactic reaction	1 (0.6)	0
Blood culture positive	1 (0.6)	0
Cardiac disorders	1 (0.6)	0
Congestive cardiac failure	1 (0.6)	0
Coronary artery disease	1 (0.6)	0
Ectopic pregnancy	1 (0.6)	0
Injury, poisoning and procedural complications	1 (0.6)	1 (0.7)
Road traffic accident	1 (0.6)	0
Soft tissue injury	0	1 (0.7)

Nervous system disorders	1 (0.6)	0
Cerebral hemorrhage	1 (0.6)	0
Cerebrovascular accident	1 (0.6)	0
Skin and subcutaneous tissue disorders	0	2 (1.3)
Contact dermatitis	0	1 (0.7)
Hidradenitis	0	1 (0.7)
Diabetic ketoacidosis	0	1 (0.7)
Deep vein thrombosis	0	1 (0.7)

AE, adverse event.

Table S3. Participant Satisfaction Survey Results

	Usual Care (n=156)	Clinical Pathway (n=153)	Difference Between Periods (95% CI)
Participants with any survey data, n (%)	119 (76.3)	124 (81.0)	-4.8 (-14.5, 5.0)
Participants with all survey questions completed, n (%)	0	0	
Wait in ED, hours			
Number	119	124	
Median (Q1, Q3)	9.0 (7.0, 10.0)	9.0 (8.0, 10.0)	
Respondents satisfied with their hospital stay			
Number (%)	63/119 (52.9)	27/119 (21.8)	31.2 (18.8, 43.5)
Median (Q1, Q3)	9.0 (7.0, 10.0)?	8.0 (7.0, 9.0)?	
Respondents satisfied with IV antibiotic therapy			
Number (%)	86/119 (72.3)	119/124 (96.0)	−23.7 (−33.3, −14.1)
Median (Q1, Q3)	9.0 (8.0, 10.0)?	10.0 (8.0, 10.0)?	
Healthcare setting preferred if treated again for a skin infection with IV, n (%)			
Outpatient care: Brief hospital visit that did not require an overnight stay	74/116 (63.8)	106/123 (86.2)	−22.4 (−33.9, −10.9)
Overnight hospital stay for one or more nights	42/116 (36.2)	17/123 (13.8)	22.4 (10.9, -33.9)
Factors contributing to preference for outpatient care, n (%)			

	Usual Care (n=156)	Clinical Pathway (n=153)	Difference Between Periods (95% CI)
Outpatient care would allow me to avoid staying in the hospital for one or more nights	52/156 (33.3)	91/153 (59.5)	−26.1 (−37.5, −14.8)
Outpatient care would allow me to return to work/school	37/156 (23.7)	44/153 (28.8)	−5.0 (−15.5, 5.4)
Outpatient care would allow me to return to performing my normal activities of daily living (caring for myself, housework, preparing meals, etc.)	48/156 (30.8)	65/153 (42.5)	−11.7 (−23.0, −0.4)
Outpatient care would allow me to return to providing care to others (family members, dependents, etc.)	27/156 (17.3)	36/153 (23.5)	−6.2 (−15.8, 3.4)
Outpatient care would be more affordable	21/156 (13.5)	38/153 (24.8)	−11.4 (−20.7, −2.0)
Outpatient care would be more convenient for me	43/156 (27.6)	64/153 (41.8)	−14.3 (−25.4, −3.1)
Outpatient care would ensure regular monitoring by healthcare providers	10/156 (6.4)	22/153 (14.4)	−8.0 (−15.4, −0.6)
Outpatient care would be administered by a skilled healthcare provider	17/156 (10.9)	27/153 (17.6)	−6.7 (−15.2, 1.7)
Other	3/156 (1.9)	1/153 (0.7)	1.3 (−1.9, 4.4)
I would not prefer outpatient care	38/156 (24.4)	14/153 (9.2)	15.2 (6.4, 24.0)
Regimen preferred if treated again for a similar skin infection with IV, n (%)			
One single IV antibiotic dose to complete your course of treatment	65/117 (55.6)	107/121 (88.4)	−32.9 (−44.4, −21.4)
One or two daily IV antibiotic doses every day for 7−14 days to complete your course of treatment	14/117 (12.0)	6/121 (5.0)	7.0 (−0.9, 14.9)

	Usual Care (n=156)	Clinical Pathway (n=153)	Difference Between Periods (95% CI)
One or two daily IV antibiotic doses for a few days, then oral antibiotics three or four times per day for the remaining 7- to 14-day course of treatment	38/117 (32.5)	8/121 (6.6)	25.9 (15.5, 36.3)
Time willing to spend receiving each IV, n (%)			
About 30 minutes or less	73/117 (62.4)	87/123 (70.7)	−8.3 (−21.1, 4.4)
About 1 hour	28/117 (23.9)	32/123 (26.0)	−2.1 (−13.9, 9.7)
About 1.5 to 2 hours	13/117 (11.1)	3/123 (2.4)	8.7 (1.5, 15.8)
About 3 hours or longer	3/117 (2.6)	1/123 (0.8)	1.8 (−2.4, 5.9)
Find value for IV antibiotic administration in outpatient setting recommended by physician, n (%)			
Definitely not	13/115 (11.3)	5/123 (4.1)	7.2 (−0.4, 14.8)
Probably not	13/115 (11.3)	8/123 (6.5)	4.8 (−3.3, 12.9)
Probably so	38/115 (33.0)	35/116 (30.1)	2.9 (−10.0, 15.7)
Definitely so	51/115 (44.3)	73/123 (59.3)	−15.0 (−28.4, −1.6)

CI, confidence interval; ED, emergency department; IV, intravenous; Q1, 25th percentile; Q3, 75th percentile

Table S4. Participant-Reported Work Productivity and Activity Impairment on Usual Care and After Implementation of a Skin and Soft Tissue Infection Clinical Pathway

Outcomes	Usual Care (n=156)	Clinical Pathway (n=153)	Difference between periods (95% CI)
Respondents reporting absenteeism from work, n/N (%)	19/58 (32.8)	18/54 (33.3)	0.6% (−18.6%, 19.8%)
Respondents reporting impairment while working, n/N %	19/53 (35.8)	13/48 (27.1)	−8.8% (−28.8%, 11.2%)
Respondents reporting overall work impairment, n/N (%)	23/53 (43.4)	18/48 (37.5)	−5.9 (−27.0%, 15.2%)
Respondents reporting non-work related activity impairment, n/N (%)	55/121 (45.5)	34/123 (27.6)	−17.8 (-30.5%, -5.1%)

CI, confidence interval

Table S5. Patient-Reported Health-Related Quality of Life Outcomes on Usual Care and after implementation of a Skin and Soft Tissue Infection Clinical Pathway

Outcomes	Usual Care (n=156)	Clinical Pathway (n=153)	Difference between periods (95% CI)
SF12 MCS at baseline			
Number	148	150	
Median (Q1, Q3)	50.1 (37.9, 56.4)	51.4 (41.8, 57.7)	−1.3
Range	16.9, 71.9	14.6, 71.4	
SF12 MCS at Day 14			
Number	121	122	
Median (Q1, Q3)	54.5 (46.0, 58.0)	55.5 (48.8, 59.1)	−1.0
Range	16.9, 71.4	12.3, 67.1	
SF-12 PCS at baseline			
Number	148	150	
Median (Q1, Q3)	40.4 (32.1, 47.6)	42.2 (33.2, 49.3)	−1.8
Range	19.0, 62.9	18.3, 63.9	
SF-12 PCS at Day 14			
Number	121	120	
Median (Q1, Q3)	48.0 (39.8, 55.4)	49.8 (46.6, 56.0)	−1.8
Range	20.5, 60.4	17.7, 61.0	

MCS, mental component summary; PCS, physical component summary; Q1, 25th percentile; Q3, 75th percentile; SF-12, Health-Related Quality of Life Medical Outcomes Study Short Form-12.
